# Clinical Pharmacokinetics of Tacrolimus in Iranian Liver Transplant Recipients

**Published:** 2014

**Authors:** Zahra Nasiri-Toosi, Simin Dashti-Khavidaki, Mohsen Nasiri-Toosi, Ali Jafarian, Hossein Khalili, Shirinsadat Badri, Sima Sadrai

**Affiliations:** a Faculty of Pharmacy, Tehran University of Medical Sciences, Tehran, Iran.; b Faculty of Pharmacy, Tehran University of Medical Sciences, Tehran, Iran.; c Imam Khomeini Hospital Complex, Tehran University of Medical Sciences, Tehran, Iran.; d Imam Khomeini Hospital Complex, Tehran University of Medical Sciences, Tehran, Iran.; e Faculty of Pharmacy, Tehran University of Medical Sciences, Tehran, Iran.; f Faculty of Pharmacy, Isfahan University of Medical Sciences, Isfahan, Iran.; g Faculty of Pharmacy, Tehran University of Medical Sciences, Tehran, Iran.

**Keywords:** Liver transplantation, Pharmacokinetics, Tacrolimus

## Abstract

Tacrolimus, a cornerstone of immunosuppressive therapy in solid organ transplantation, has a narrow therapeutic range with considerable inter-individual and intra-individual pharmacokinetic variability. To date, there is no information on the pharmacokinetics of tacrolimus in Iranian liver transplant recipients. This study was designed to determine pharmacokinetic properties of orally administered tacrolimus in Iranian adult liver transplant recipients.

Tacrolimus doses and steady state whole blood trough concentrations as well as patient demographic and clinical data were obtained retrospectively using the 30 included patients’ medical records. Pharmacokinetic parameters were estimated by using a nonlinear mixed effect model program (Monolix version 3.1). Absorption rate constant was fixed at two hours^-1^. Drug apparent clearance (CL/F), apparent volume of distribution (Vd/F), and elimination half life (t_½β_) were calculated.

The administered dose of tacrolimus to the patients ranged from 0.02 to 0.14 mg/Kg/day. Tacrolimus blood trough concentrations varied widely within the range of 1.8 to 30 ng/mL. The mean values of CL/F, Vd/F, and t_½β_ were found to be 9.3 ± 0.96 L/h, 101 ± 29 L, and 7.5 hours, respectively.

The pharmacokinetics of tacrolimus was highly variable among our patients. CL/F, Vd/F, and t_½β_ of tacrolimus in this study were comparable to reported values from Italian heart transplant patients but somewhat different from reported ones from other solid organ transplant populations.

## Introduction

Tacrolimus, a potent immunosuppressive agent, has been used since 1994 as a valuable alternative to cyclosporine for the prophylaxis of acute organ rejection after liver transplantation. It is also effective in preventing graft rejection in heart, small bowel, and kidney transplant recipients. (1) As other immunosuppressive agents (2), tacrolimus has a narrow therapeutic range with considerable inter-individual and intra-individual pharmacokinetic variability that makes it impossible to give a standard dosing regimen to all adults to achieve goal blood concentrations. To prevent transplant rejection, immune suppressants should be administered in adequate amounts. In contrast, excessive immune suppression can lead to serious toxicities and infections; hence, therapeutic drug monitoring (TDM) and dosage individualization based on patient’s pharmacokinetics are recommended to optimize treatment outcome (3).

A number of studies have evaluated the pharmacokinetic properties of tacrolimus. Based on clinical experiences, it has been shown that tacrolimus blood trough concentrations are significantly related to clinical endpoints. Accordingly, in order to maintain the graft and prevent drug toxicity, tacrolimus blood trough concentrations of 10 to 20 ng/mL during the first month after liver transplantation; 5 to 15 ng/mL, during the subsequent 2 months, and 5 to 10 ng/mL thereafter, were targeted in most protocols (4).

To date, there is no information on the pharmacokinetics of tacrolimus in Iranian liver transplant recipients. In this paper we reported the first estimation of tacrolimus pharmacokinetic parameters based on the findings of small sample of Iranian adult liver transplant recipients.

## Experimental

*Patients and data collection*

Thirty adult patients who underwent liver transplantation at Imam Khomeini Hospital Complex affiliated to Tehran University of Medical Sciences included in this study. Research protocol was approved by ethics committee of Tehran University of Medical Sciences. Demographic, Clinical and laboratory data were obtained from the patients' medical records. None of the patients were on any drug, which is known to interfere with tacrolimus metabolism during the study period. 

*Drug administration*

All patients received oral tacrolimus as a part of regimen that included mycophenolate mofetil and prednisolone as well. Therapy was generally initiated at a dose of 0.1 mg/Kg twice daily. Subsequent doses were adjusted based on the clinical status of the patient and to maintain tacrolimus trough blood concentrations within the target range of 10 and 20 ng/mL in the first three months post-transplantation and between 5 and 15 ng/mL thereafter.

*Therapeutic drug monitoring and pharmacokinetic parameters estimation*

Blood samples for determining whole blood tacrolimus concentration were collected after the patients were at steady state before the administration of morning dose of the drug. Subsequent sampling occurred periodically at outpatient visits. Trough samples were also taken if any signs and symptoms of rejection were observed or as needed to manage any suspected adverse event. Tacrolimus whole blood concentrations were measured by Dade Behring Emit 2000 Tacrolimus Assay kit according to the instructions of the manufacturer. Pharmacokinetic parameters were estimated by using a nonlinear mixed effect model program (Monolix version 3.1) (5). Parameters were estimated by minimizing ‘maximum likelihood estimator’ of the parameters. A constant model was used to describe the residual variability (6). Because no data from the absorption phase was available, absorption rate constant (k_a_) was fixed at two hours^-1^ as previously reported in a population pharmacokinetic analysis (7). Blood concentrations of tacrolimus were fitted to a one-compartment model with first-order elimination (8). Data were presented as mean ± standard error.

## Results

*Clinical observations*

This study was performed on 30 adult patients (15 males and 15 females). Patients’ characteristics including demographic and pharmacokinetic data have been summarized in Table 1. Patients required transplantation because of liver cirrhosis caused by autoimmune liver disease (ten patients), hepatitis B infection (seven patients), cryptogenic liver cirrhosis (six patients), hepatitis C infection (five patients), and Wilson’s disease (two patients). Two hundred and twenty six blood samples were obtained from 30 patients. On average we had seven blood samples from each patient. No transplant rejection has been occurred among our patients.

**Table 1:** Characteristics of study patients

**Characteristic**	**Data**
**Demographic data**	
Age (years)	38.0 ± 12.4
Sex (male/female)	15/15
Weight (kg)	66.1 ± 12.6
Height (cm)	166.4 ± 7.8
**Pharmacokinetic data**	
Dose (mg/kg/day)	0.062 ± 0.024
Concentration (ng/mL)	11.1 ± 6.8

Data are presented as Mean ± SD

*Pharmacokinetic results*

The administered dose of tacrolimus to these patients was 0.06 ± 0.02 mg/Kg/day. Apparent clearance based on whole blood concentration after oral administration (CL/F), apparent volume of distribution based on whole blood concentration after oral administration (V/F) and half-life (t_1/2_) were 9.3 ± 0.96 L/h, 101 ± 29 L, and 7.5 hours, respectively.

## Discussion

This is the first report of pharmacokinetic parameters of tacrolimus in Iranian adult liver transplant recipients. Since the pharmacokinetic parameters of tacrolimus are a function of the biological matrix analyzed (plasma or whole blood) and the analytical methods, it is difficult to draw an exact comparison between our results and those reported in other studies. It is important to mention that although plasma through concentrations of tacrolimus closely reflect the pharmacologically active moiety of the drug, whole blood appears to be the preferred matrix for tacrolimus therapeutic drug monitoring (9). All of the articles which we compared our results with, were obtained based on whole blood concentration.

The pharmacokinetics of tacrolimus was highly variable among our patients. The calculated value of CL/F was 9.3 ± 0.96 L/h which is somewhat less than those reported in previous studies in Singaporean adult liver recipients (14.1 L/h) (10) and Italian heart transplant recipients (0.21 ± 0.08 L/h/Kg). (11) This value was much lower than the reported tacrolimus CL/F in adult kidney transplant recipients (33 ± 11.3 L/h) (12), Australian adult liver transplant recipients ((26.5 ± 8.2 L/h) (13) and (21.3 ± 8.4 L/h) (14)). 

The obtained apparent volume of distribution of the orally administered tacrolimus in this study was 101 ± 29 L, which is comparable to the previous study in Italian heart transplant recipients (2.4 ± 0.8 L/Kg) (11). This value was lower than reported values from adult liver transplant recipients (316.1 L) (13), Asian liver transplant recipients (217 L) (10), and Australian adult liver transplant recipients (399 ± 185 L) (13).

The calculated value of elimination half life in the present study was 7.5 hours, which is comparable to the previous study in Italian heart transplant recipients (8.7 ± 3.5 h) (11). This value was lower than adult kidney transplant recipients (48.1 ± 121.1 h) (12).

There are a number of reasons for our results. First, the binding of tacrolimus to erythrocytes is expected to limit its clearance and appears to be a major factor accounting for the large interpatient variability in the pharmacokinetics of tacrolimus. (1) Second, we adopted a nonspecific assessment method, which measures some of the tacrolimus metabolites and may overestimate the concentration of tacrolimus; however, this was the only available method for the measurement of tacrolimus blood concentration in Iran.

## Conclusion

Given that tacrolimus has a narrow therapeutic index, it is important to monitor the blood concentrations of this drug in transplant patients. This study was conducted retrospectively in a small patient population with the use of data that were available as part of the routine patient care recorded in the medical notes and transplant flowcharts. Apparent clearance (CL/F), apparent volume of distribution (Vd/F), and elimination half life (t_½β_) of tacrolimus in this study were comparable to reported values from Italian heart transplant patients but somewhat different from reported ones from other solid organ transplant populations. 

Of course, large prospective population pharmacokinetic studies are needed to determine the more accurate and complete pharmacokinetic parameters of tacrolimus in Iranian population that seems to be somewhat different with other populations. So, tacrolimus dose might be much accurately adjusted accordingly in this transplant recipient population.
